# Talking about Relations: Factors Influencing the Production of Relational Descriptions

**DOI:** 10.3389/fpsyg.2016.00103

**Published:** 2016-02-09

**Authors:** Adriana Baltaretu, Emiel J. Krahmer, Carel van Wijk, Alfons Maes

**Affiliations:** Tilburg Center for Cognition and Communication, Tilburg UniversityTilburg, Netherlands

**Keywords:** reference production, relatum, spatial position, animacy, perceptual salience, attention capture, relational descriptions, referring expressions

## Abstract

In a production experiment (Experiment 1) and an acceptability rating one (Experiment 2), we assessed two factors, spatial position and salience, which may influence the production of relational descriptions (such as “the ball between the man and the drawer”). In Experiment 1, speakers were asked to refer unambiguously to a target object (a ball). In Experiment 1a, we addressed the role of spatial position, more specifically if speakers mention the entity positioned leftmost in the scene as (first) relatum. The results showed a small preference to start with the left entity, which leaves room for other factors that could influence spatial reference. Thus, in the following studies, we varied salience systematically, by making one of the relatum candidates animate (Experiment 1b), and by adding attention capture cues, first subliminally by priming one relatum candidate with a flash (Experiment 1c), then explicitly by using salient colors for objects (Experiment 1d). Results indicate that spatial position played a dominant role. Entities on the left were mentioned more often as (first) relatum than those on the right (Experiments 1a–d). Animacy affected reference production in one out of three studies (in Experiment 1d). When salience was manipulated by priming visual attention or by using salient colors, there were no significant effects (Experiments 1c, d). In the acceptability rating study (Experiment 2), participants expressed their preference for specific relata, by ranking descriptions on the basis of how good they thought the descriptions fitted the scene. Results show that participants preferred most the description that had an animate entity as the first mentioned relatum. The relevance of these results for models of reference production is discussed.

## 1. Introduction

Human speakers have a rich repertoire for referring to objects in visual scenes. For example, if you want to buy a ball from the toy store, the shop assistant could help you find it among other balls by referring to intrinsic attributes (e.g., color, *the red ball*) or extrinsic ones (e.g., location, *the ball between the doll and the train*). An object's location can be described in relation to one's body and to other objects or to environmental features (Levinson, 1996). In the current work, we focus on referential choices when describing external relations (Levinson, 2003; Tenbrink, 2011) where an object is the target, while other object(s) serve as the relatum. The target is sometimes referred to as the locatum, figure or located object, whereas the relatum is also known as ground, reference location or landmark. In the previous example, the ball represents the target and it is described in relation to two relata objects, the doll and the train.

Compared to intrinsic attributes (such as color), there are few studies in the referring expressions generation field analyzing how extrinsic attributes (such as location) are used in order to refer unambiguously to a target object (for a review, see Krahmer and Van Deemter, 2012). When talking about location, speakers describe where the target object is positioned in space. Far from being a trivial feature, space is a pervasive dimension in language and cognition. For example, we map time onto space (e.g., Boroditsky, 2000), make use of space in gestures (e.g., Gentner et al., 2013), in discourse (e.g., Lakoff and Johnson, 2008), and in actions (e.g., Kirsh, 1995). Crucially, humans employ location in a meaningful way in different forms of descriptions and visualizations. It is natural to refer to an object's location in a variety of situations, thus anchoring the conversation topic in the spatio-temporal context (Levelt, 1993, p.51). Such situations are, among other things, route direction production, interaction with conversational agents, visual communication (e.g., maps and graphs) within various disciplines (e.g., architecture, geosciences, engineering, etc., for a review, see Tversky, 2011).

Pervasive use of spatial relations in real life communication makes it necessary to develop referring expression generation algorithms that can handle such reference. These algorithms (e.g., the Incremental Algorithm, Dale and Reiter, 1995; the Graph-Based Algorithm, Krahmer et al., 2003) have a key role in natural language generation, enabling machines to make informed choices and to refer to objects in a more human-like manner (van Deemter et al., 2012; Gatt et al., 2014; Dos Santos Silva and Paraboni, 2015). Though we know little of the situations when relational descriptions are spontaneously produced and preferred over intrinsic attributes, there are communicative contexts in which relations are an efficient and relevant strategy [like in route directions or in scenes with many (similar) objects]. Recent studies have shown that speakers often produce relational descriptions in order to single target objects out of other objects in a visual scene (Clarke et al., 2013a; Kazemzadeh et al., 2014). When both intrinsic and extrinsic attributes are available, people tend to mention location even when this attribute is not necessary for producing a unique object description (Viethen and Dale, 2008). Listeners seem to benefit from this type of reference as well (Arts et al., 2011; Paraboni and van Deemter, 2014). Currently, spatial relations represent a major challenge for referring expressions generation algorithms, as we know little about the situations in which speakers employ them in the context of identification. To further develop these algorithms, more input from studies on human reference is needed.

In this series of studies, we focus on human reference production in spatial relational descriptions. In visual scenes, several entities can be in the proximity of the target and each one of them could be a potential relatum. In our previous example, the shop assistant could either refer to the target as, for example, *the ball in front of the doll* (using a single relatum) or *the ball between the doll and the train* (using two relata). In the first description, which we call *the single-relatum formulation*, the question is what causes speakers to mention one of the objects. In the second strategy, *the two-relata formulation*, we question what causes speakers to mention one of the objects as first relatum. In the two-relata formulation, we consider important the order in which entities are mentioned. Word order choices have been previously suggested to reflect speaker's referential preferences (Goudbeek and Krahmer, 2012) and the ease with which these entities are processed (Bresnan et al., 2007; Onishi et al., 2008; Jaeger and Tily, 2011).

While the study of spatial relations in the field of referring expression generation is a topic largely unexplored, in the field of spatial cognition there have been numerous studies concerned with principles that govern relatum object selection (e.g., Barclay and Galton, 2008; Miller et al., 2011; Barclay and Galton, 2013), the choice of adequate spatial prepositions based on geometric and functional characteristics of the objects (e.g., Carlson-Radvansky et al., 1999; Coventry and Garrod, 2004) and the influence of frames of reference on relatum selection (e.g., Carlson-Radvansky and Radvansky, 1996; Levinson, 2003; Taylor and Rapp, 2004; Tenbrink, 2007). Various factors might affect the selection of a relatum object. Compared to target objects, relata are described as larger, closer to the target, geometrically more complex (Barclay and Galton, 2013) as well as more familiar, expected, more immediately perceivable (Talmy, 2003).

In this series of studies, we seek to investigate speakers' referential choices, aiming thereby to provide further insight for REG algorithms. Most studies mentioned above focus on the problem of localization, as opposed to identification (Tenbrink, 2005; Dos Santos Silva and Paraboni, 2015). In localization tasks speakers are restricted to refer to already agreed upon objects (e.g., the target and relatum are given and a priori labeled as, for example “cup"), based solely on their spatial locations. On the other hand, freely producing a referring expression (like “the cup between the plate and the kettle") is a matter of choosing attributes of the target (including its spatial position), to help the addressee identify a target object out of several candidates. Comparisons between identification and localization tasks have been previously addressed (Tenbrink, 2005; Moratz and Tenbrink, 2006; Vorwerg and Tenbrink, 2007). In general, descriptions seem to be more detailed when the target needs to be localized, rather than identified. Factors to influence reference production (e.g., spatial biases, conceptual and visual salience) have been addressed to a lesser extent.

It is generally assumed that if an object is salient, it can grab visual attention, and thus is likely to be selected and mentioned as relatum (Beun and Cremers, 1998; Tversky et al., 1999). A number of visual factors have been identified as important cues for salience, such as size, color, orientation, foregrounding, animacy (for a review, see Wolfe, 1994; Parkhurst et al., 2002; Kelleher et al., 2005; Coco and Keller, 2015), but little is known about how these and other cues influence reference production. The goal of the current research is to examine two factors previously shown to influence language production and comprehension in general, yet understudied in reference production: spatial position and salience.

### 1.1. Spatial position: A left-to-right preference?

Referring to a relatum may be influenced by a factor present in any visual scene: the position of the object in the scene. Different types of evidence suggest there might be a bias to choose objects placed in specific locations. Speakers choose and mention spatially aligned and proximate objects as relata (e.g., Craton et al., 1990; Hund and Plumert, 2007; Viethen and Dale, 2010; Miller et al., 2011). Yet, when several objects are in the vicinity of the target, all similarly aligned, would spatial features continue to influence reference production? We assume that it does, and objects on the left of the target would be mentioned more often as relatum than objects on the right. This prediction is based on findings from various disciplines as follows.

The speaker's attention might be guided by different factors toward specific regions of the scenes. One line of research suggests that oculomotor biases (the amplitude and direction of saccades—movements of the eye between fixation points) are an important predictor for the location where speakers initially direct their attention (e.g., Tatler and Vincent, 2009; Kollmorgen et al., 2010). One well known, image independent bias is the tendency to look at the center of visual stimuli during image exploration (for a review, see Clarke et al., 2013a). Besides this bias, there is also evidence for a horizontal spatial bias (sometimes referred to as “pseudoneglect”). People initially execute more often leftward than rightward saccades, irrespective of the content of the image, across different tasks (free viewing, memorization, scene search, Foulsham et al., 2013; Ossandón et al., 2014). This asymmetry seems to affect memory, with left positioned objects being better remembered than right positioned ones (Dickinson and Intraub, 2009).

Converging evidence comes from cross-cultural psychology research where the left-to-right bias is considered to be a result of the scanning routines employed during reading and writing. The directionality of the language system has an impact on visual attention, memory, and spatial organization (Chan and Bergen, 2005). For instance, when participants with a left-to-right language system (in this case: French) were asked to mark the middle of a straight line, they usually misplaced the mark to the left of the objective middle, while participants with a right-to-left language system (Hebrew) misplaced the mark to the right (Chokron and Imbert, 1993). Such a bias is shown from a young age in graphical representations of spatial and temporal relations (Tversky et al., 1991). This implies that, at least in western cultures, people “read” visual scenes from left to right and that the left-to-right bias might be a habit acquired by systematically using a language system.

The directionality of the writing system seems to affect cognitive linguistic processes. In picture description tasks, speakers of left-to-right languages tend to scan, describe and remember items from left to right (Taylor and Tversky, 1992; Meyer et al., 1998). Speakers of different writing systems show different patterns of sentence production. For example, in a sentence-picture matching task, speakers of a language with a left-to-right (in this case: Italian) system tended to choose visual scenes with the agent placed on the left of the patient, those of a language with a right-to-left system (Arabic) preferred scenes with the agent placed on the right of the patient (Maass and Russo, 2003; Chan and Bergen, 2005). Not only the writing system, but also the dominant frame of reference of the language, might affect the order in which speakers refer to entities in visual scene. For example, when using a relative frame of reference, to perceive that something is “on the left,” the speaker would project his viewpoint onto the scene (Levinson, 2003). Bilingual speakers of Spanish (relative frame of reference) and Yucatec (no dominant frame of reference), show a bias to start with the left object in the scene when using Spanish, but not when doing this task in Yucatec (Butler et al., 2014).

The left-to-right bias was also observed in clinical populations. Participants suffering from agrammatism, an aphasic syndrome, presented a similar left-to-right bias both in language production (describing visual scenes) and comprehension (matching sentences with pictures, Chatterjee, 2001). In addition, studies in the psychology of art suggest that reading habits influence visual preferences: participants preferred pictures possessing the same directionality as their reading system (Chokron and De Agostini, 2000).

**Figure 1:**
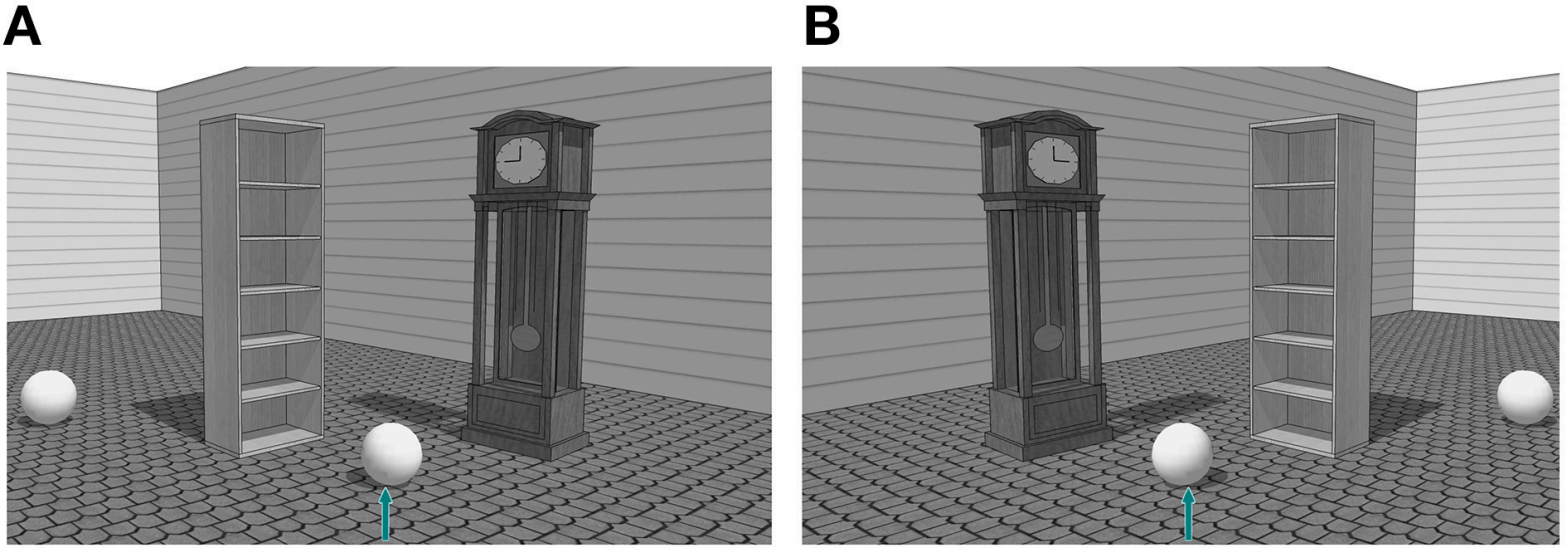
**Experimental stimulus with inanimated object (bookshelf) on the left (A) and the right (B) of the target**.

Given the evidence for a left-to-right bias, there might be a tendency for speakers to mention relata based on their position in the scene. For example, in Figure 1, speakers could refer to the target as in (a) *the ball in front of the bookshelf*, (b) *the ball in front of the clock* or (c) *the ball between the bookshelf and the clock*. These three descriptions were considered valid for identification and classified in two formulation preferences: the single-relatum formulation (descriptions a and b) and the two-relata formulation (description c). When only one object was mentioned, we considered it to reflect the speakers' preference for a relatum candidate. In case both entities were mentioned, we took into account the order of mentioning. If a left-to-right bias plays a role in reference production, we expect entities left of the target to be mentioned more often as relatum (as in *a*) or mentioned more often as the first relatum (as in *c*). However, a spatial bias, might not be the sole factor that influences relatum reference. In the following section, we review evidence for other factors that potentially contribute to the salience of relatum candidates.

### 1.2. Salience

Salience is generally considered an important factor for reference production. The objects' salience captures visual attention and entities in focus of attention during utterance planning have higher chances of being mentioned (Beun and Cremers, 1998; Gleitman et al., 2007). In the present study, salience (the property of being noticeable or important) is operationalized in two ways.

We distinguish between conceptual and visual salience. By conceptual salience, we refer to the ease of activation of mental representations caused by knowledge-based conceptual information (or “accessibility” in Bock and Warren, 1985; Ariel, 1990). There are several properties of the referent that contribute to its conceptual salience (e.g., linguistic properties, such as the syntactic position a referent occupies; context, such as the preceding discourse; intrinsic properties, such as animacy, etc.). In this study we focus on animacy: whether an entity is conceptualized as living or not (Vogels et al., 2013; Coco and Keller, 2015). In contrast, by visual salience we touch on two different aspects: perceptual salience and visual priming. By perceptual salience, we refer to bottom-up, stimulus-driven signals that attract visual attention to areas of the scene that are sufficiently different from the surroundings (Itti and Koch, 2001). For example, a perceptually salient object is an object that has a unique color compared to the rest of the scene. Moreover, entities can become salient when visual attention is guided toward them, for example by using attention priming techniques (Gleitman et al., 2007). Below we discuss these types of salience in more detail.

#### 1.2.1. Conceptual salience

Animacy is a basic conceptual feature of objects and there are reasons to believe that it may affect the production of relational descriptions. First, animacy has been shown to influence the allocation of visual attention. Humans prioritize the visual processing of animate objects over inanimate ones (Kirchner and Thorpe, 2006; New et al., 2007; Fletcher-Watson et al., 2008). Both visual representations of the face and the human body have the ability to capture the focus of attention, even when attention is occupied by another task (Downing et al., 2004). Compared to inanimate objects, animate entities are more likely to be fixated and named (Clarke et al., 2013b; for a review, see Henderson and Ferreira, 2013).

Second, animacy is known to play a key role in reference production (Clark and Begun, 1971; McDonald et al., 1993). Animate entities are conceptually highly accessible, thus, retrieved and processed more easily than inanimate entities (Prat-Sala and Branigan, 2000). This can influence word ordering, as there is a strong tendency for the animate entities to occupy more prominent syntactic positions (e.g., in the beginning of a structure) and grammatical functions (e.g., subject role) (e.g., Bock et al., 1992; McDonald et al., 1993; Prat-Sala and Branigan, 2000; Branigan et al., 2008). Additionally, compared to inanimate referents, animates are mentioned more frequently and are more likely to be pronominalized (e.g., Fukumura and van Gompel, 2011).

Given that utterance planning is influenced by conceptual factors and that animacy has a privileged role in language production, we could expect animate entities to be mentioned as relatum (or as first relatum) more often than inanimate ones due to their conceptual salience, irrespective of their position with respect to the target. In general, there is little evidence that animacy could influence relatum choice. The few studies that looked at this, directly or indirectly, do not present a consistent picture. Under specific circumstances, de Vega et al. (2002) report that relata can be animate, but only when included in a construction using the preposition *behind* [the animate entity]. Congruent evidence was found in a large English corpus of referring expressions elicited with complex naturalistic scenes. Speakers were shown an image with an outlined object and provided with a text box in which to write a referring expression. When speakers decided to produce spatial relational descriptions, the most frequent relata objects were people and some entities positioned in the background, such as trees and walls (Kazemzadeh et al., 2014). Taylor et al. (2000), however, argue that animate entities should be disfavored as relata due to their mobility.

#### 1.2.2. Visual salience

Reference production was shown to be sensitive to both visual priming (e.g., a short flash at the target location, Gleitman et al., 2007) and perceptual salience cues, such as uniquely colored objects (Pechmann, 1989; Belke and Meyer, 2002).

Priming participants' initial gaze to a specific area of a scene has been claimed to influence grammatical role assignment and word order (Gleitman et al., 2007). When visual attention is guided toward it, an object is more likely to be mentioned in the beginning of a description or relation (in a prominent grammatical role, such as subject, or in a prominent position in the utterance). As far as we know, no studies looked into effects of attention manipulation on spatial relational descriptions. Reference production can be influenced by very basic, implicit attention-grabbing cues. Gleitman et al. (2007) report that presenting a flash shortly before displaying a scene, systematically redirected the gaze of the participants to the location of a specific object (occurring at the location of the flash), which later received a privileged position in the sentence structure. The short duration of the flash ensured that participants remained unaware of the manipulation, while their gaze was attracted to the cued location in an implicit manner.

A similar approach has been used for the study of spatial relational descriptions (*X is left of Y*). Forrest (1996) drew speakers' attention to the location of an object, prior to the scene presentation. Unlike Gleitman et al. (2007), she used an explicit visual cue, a flash that lasted long enough to be noticed by the participants. This explicit visual cue influenced speakers' description as well: the object which appeared in the primed location generally received a more prominent place in the beginning of the sentence.

Apart from priming, properties of the stimulus may play a crucial role in guiding the eyes. Perceptual salience is a factor known to influence visual attention (for review, see Tatler et al., 2011) and reference production (Myachykov et al., 2011; Clarke et al., 2013b; Coco and Keller, 2015). Perceptual salience is a characteristic of parts of a scene (objects or regions), that appear to stand out relative to their neighboring parts and there are several models to account for this phenomenon (for a review, see Borji and Itti, 2013). Most models use image features, such as color, contrast, orientation and motion and make center-surround operations to compare the statistics of image features at a given location to the statistics in the surrounding area (Borji and Itti, 2013).

Among these features, color has been shown to capture visual attention (Folk et al., 1994; Parkhurst et al., 2002), irrespective of the observers' task (Theeuwes, 1994). In general, color enhances object recognition (for a review, see Tanaka et al., 2001) and uniquely colored items are detected faster than other objects in the scene, regardless of the amount of distractors (Treisman and Gelade, 1980; D'Zmura, 1991).

In general, scholars suggest that explicit perceptual features (such as color, size, shape) may contribute to relatum selection (e.g., Barclay and Galton, 2008), yet there are almost no experimental studies which try to disentangle the effects of these features. Regarding the influence of color on relatum selection and reference, prior results are equivocal (Miller et al., 2011, Viethen et al., 2011). Yet, in reference production studies, color is probably the attribute mentioned most frequently. In reference tasks, color is considered to have a high pragmatic value (Belke and Meyer, 2002; Davies and Katsos, 2009). Speakers mention it even when this information is not needed for identification (Koolen et al., 2011; Westerbeek et al., 2015). In complex scenes, reference to both target and relatum objects is affected by perceptual salience (a composite measure of color and other low level visual features), visual complexity (clutter), size and proximity (Clarke et al., 2013a). Clarke et al. (2013a) note that relatum objects were chosen based on their size and saliency; while references to less salient target objects included a higher number of relata.

Moreover, the order in which objects are mentioned in a relational description may be sensitive to perceptual salience as well. In visual domains, speakers can mention target and relatum objects in different orders. Elsner et al. (2014) report that speakers employed complex word orders such as starting with (a) the target, (b) the relatum or by giving information about the target in multiple phrases intertwined with relatum references. For example, if the target was a person (target in **bold**, relatum in *italics*), speakers could say (a) **man** closest to *the rear tyre of the van*, (b) near *the hut that is burning*, there is **a man holding a lit torch in one hand, and a sword in the other** or (c) there is **a person standing** in *the water* **wearing a blue shirt and yellow hat** (Elsner et al., 2014, p. 522). These relations were more likely to start with the perceptually salient object.

Given these findings, we could expect objects to be mentioned as (first) relatum if they are placed in a cued location or if they are perceptually salient.

### 1.3. The current studies

Spatial position (left-to-right bias), conceptual salience (animacy), and visual salience (attention capture cues or scene based perceptual cues) all influence what is being looked at (Kollmorgen et al., 2010) and possibly mentioned (Coco and Keller, 2015). We study if and to what extent these factors influence referential choices in spatial relational descriptions.

This paper presents two experiments consisting of several parts that test the influence of these factors on relatum reference in an identification task. In Experiment 1a, we started by determining if there was a spatial bias when mentioning a relatum. We start with a basic language elicitation task that did not include any experimental factors. Its purpose was to check for a left bias in reference production. In this language elicitation task, we manipulated the position of two inanimate relatum candidates. Entities placed on the left of the target were expected to be mentioned as (first) relatum more often than those placed on the right. We took spatial position as a baseline and continued investigating the effect of salience on referential choices. Conceptual salience was manipulated by adding one animate entity in each scene (Experiment 1b). Animate entities were expected to be preferred as relatum. Visual salience was manipulated by priming attention toward a relatum candidate with a short flash (Experiment 1c) or explicitly with a unique color (Experiment 1d). Salient entities were expected to be preferred as relatum. Additionally, the listeners' preference for relata was tested, by asking participants to rank relational descriptions starting with the one that, according to them, “best fits” the scene (Experiment 2). Descriptions that have an animate entity as (first) relatum were expected to be ranked higher.

We explored these predictions across a production experiment (four parts) and in an acceptability rating experiment, and in doing so some factors may be included in several parts of these experiments (for example, the effect of spatial position is analyzed in Experiments 1, 2, animacy in Experiments 1b–d and in Experiment 2, visual salience in Experiments 1c–d). Whether speakers mentioned the left entity as (the first) relatum was tested by comparing the chance of naming the left item with random chance (0.50) using an one-sample *t*-test and possible interactions between the experimental factors were evaluated using analysis of variance (ANOVA) tests^1^.

Finally, the current studies were carried out in accordance with the recommendations of APA guidelines for conducting experiments, the Netherlands Code of Conduct for Scientific Practice and the Code for Use of Personal Data in Scientific Research (KNAW). The studies were approved by the ethics committee at Tilburg University and all participants gave written consent to the use of their data.

## 2. Experiment 1—reference production

### 2.1. Experiment 1a—position

#### 2.1.1. Participants

Thirty native Dutch undergraduates from Tilburg University participated in this study for partial course credits. Data from four speakers were discarded on the basis of task misunderstanding. The final sample consisted of 26 participants (11 female, mean age 20.19).

#### 2.1.2. Materials

The stimuli consisted of 48 grayscale scenes (12 experimental stimuli). The experimental stimuli scenes included a target item marked with an arrow (a ball), a distractor object (a ball identical to the target) in order to prevent an easy identification strategy using type only, and two relatum candidates (both inanimates). These items were eight everyday objects (such as wardrobes), easily identifiable, with a clear front/back axis and of roughly equal size, randomly coupled in pairs (see Figure 1). Filler stimuli were used to have a larger visual diversity (they included both inanimate and animate objects) and to allow participants to use a wider range of identification strategies (type, location and size). All the objects (8 animate and 8 inanimate) were pretested with a group of 10 participants, who were presented with pictures similar to the ones used in this study. They had to name the inanimate objects, as well as the gender and profession of animate objects. An inanimate object was included in the experimental stimuli if (1) it was referred to with the same noun in a minimum of 50% of the cases, and (2) if the other nouns used to refer to it, were compound nouns such as in “kast”–“ladenkast” (drawer). An animate object was chosen if (1) the character's gender was recognized in all cases and (2) if the character's profession was recognized in 80% of the cases. The scenes were created using Google SketchUp 8 (3D Warehouse library).

#### 2.1.3. Procedure

Participants were instructed to verbally refer to an object marked with an arrow in such a way that the next participant (a fictitious listener) could draw the arrows on a new set of identical pictures (language: Dutch). The goal of this instruction was to avoid participants to produce ambiguous references (for a similar procedure see Koolen et al., 2011; Clarke et al., 2013a). Participants saw each entity in three different pictures, paired every time with a different object. The materials were divided across two presentation lists, so that each participant would see each object combination only once. The position of each object and the position of the distractor ball were individually counterbalanced (half of the times they appeared on the left of the scene and half of the times on the right of the scene). Descriptions such as *the ball in front of me* or *the ball on the left* were discouraged, by telling the speaker that the listener would receive the same image, but that it might be in a mirror version. The picture remained on the screen until the participant produced a description and pressed a button to continue. Each experimental trial was followed by 3 filler trials to prevent a carry-over effect. The study started with 3 practice trials followed by 48 experimental trials and lasted approximately 10 min.

#### 2.1.4. Results and discussion

We collected 312 descriptions (26 participants ^*^ 12 experimental stimuli). Participants were found to use one of two possible formulations: either mentioning a single relatum (e.g., *the ball in front of the bookshelf*) or both (e.g., *the ball in between the bookshelf and the clock*). In all the studies of Experiment 1, the participants were grouped based on their preference for the single-relatum or the two-relata formulation strategy. Some participants systematically used a single formulation strategy, while others used both. The grouping threshold was set by inspecting the distribution of the two-relata formulation in Experiment 1. The distribution appeared to be bimodal: one group had a score of maximum 100% (down to 80); the other group had a score of maximum 40% (down to 0). Every participant with a score of 80 or more was considered to opt for a two-relata formulation and all the other for a single-relatum formulation.

In Experiment 1a participants were found to use a single-relatum formulation (*N* = 1 participant, not analyzed further due to small sample size) or a two-relata formulation (*N* = 25 participants). Whether speakers mentioned the left entity as the first relatum was tested by comparing the chance of naming the left item with random chance (0.50) using an one-sample *t*-test. Speakers mentioned the left entity as first relatum 59% of the time (95% CI [0.525; 0.659], *SD* = 0.16). This result was statistically significant [*t*_(24)_ = 2.857, *p* = 0.009; *d* = 0.57].

The results showed a left bias in reference production, however there was only a small preference in starting with the left entity. This leaves room for other factors that could influence reference. Thus, in Experiments 1b–d, we added three experimental factors that contribute to the entity's salience, making the entities “stand out” in the scene.

### 2.2. Experiment 1b—conceptual salience: Animacy

#### 2.2.1. Participants

Fifty three native Dutch undergraduates from Tilburg University participated in this study as speakers for partial course credits. Due to technical problems, speech data of four participants were not analyzed; the final sample included 49 participants (11 males, mean age 21.2 years).

#### 2.2.2. Materials

**Figure 2:**
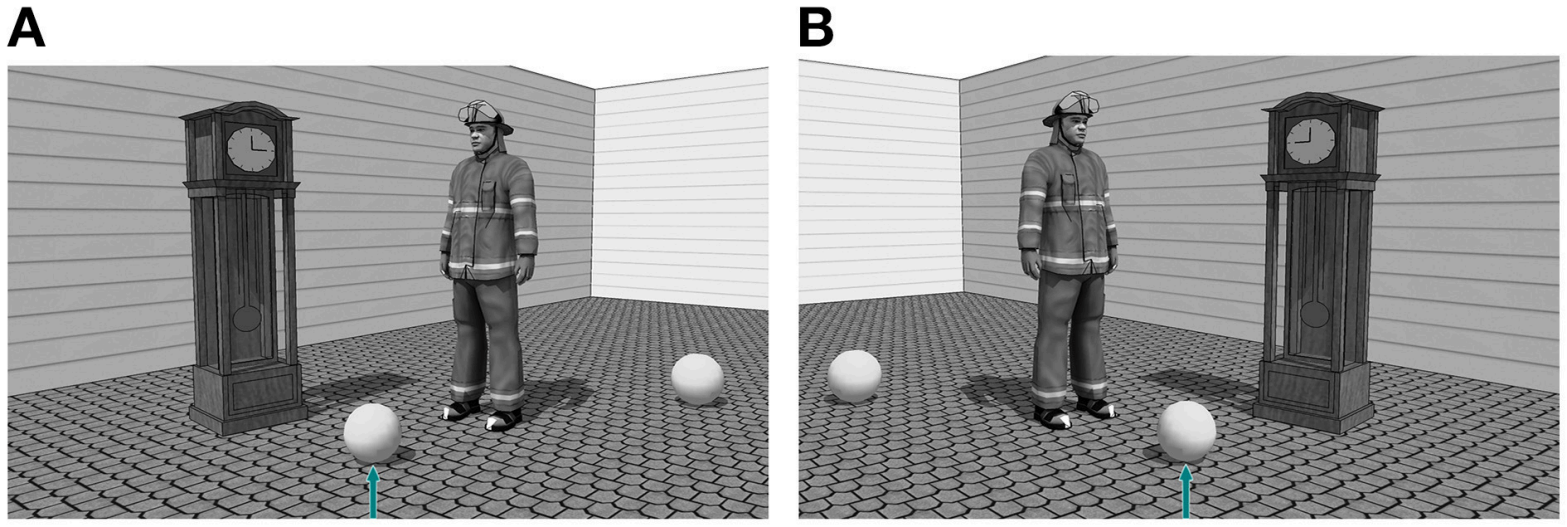
**Experimental stimulus with animated object (firefighter) on the right (A) and the left (B) of the target object**.

The stimuli consisted of 96 grayscale scenes (24 experimental stimuli). For these scenes, we used the same animate and inanimate objects described in Experiment 1a. The experimental stimuli consisted of a target and a distractor ball and two relatum candidates, one animate and one inanimate object of roughly equal size (see Figure 2). From 64 possible animate–inanimate combinations, 24 couples were randomly chosen. Filler stimuli were similar to the ones used in Experiment 1a.

#### 2.2.3. Procedure

As in Experiment 1a.

#### 2.2.4. Results and discussion

Speakers produced 1176 descriptions (49 participants ^*^ 24 experimental stimuli). Participants were found to use one of two possible formulations: either mentioning a single relatum (*N* = 12) or both relata (*N* = 37). Whether speakers mentioned the left entity as the first relatum was tested by comparing the chance of naming the left item with random chance (0.50) using an one-sample *t*-test. The chance of mentioning the left entity as first relatum was 59% [two-sided 95% CI [0.55, 0.64], *SD* = 0.17, *t*_(47)_ = 3.91, *p* < 0.001, *d* = 0.75].

Whether animacy overruled the left bias was tested with an ANOVA test, having Position of the Animate in the scene (2 levels: animate left, animate right) as a within subjects factor, and Participant Formulation Preference (2 levels: single-relatum, two-relata) as a between subjects factor. The ANOVA test revealed no statistically significant effect of Position of the Animate (*F* < 1) or of Participant Formulation Preference (*F* < 1) and no interaction between these factors (*F* < 1).

These results suggest that animacy did not influence descriptions. The responses were not affected by word frequency: 90% of the participants referred to the animate entity using highly frequent words such as *de vrouw* / *de man* (the woman / the man). However, the position of the entity was found to affect reference to a greater extent, with left entities being more likely to be mentioned as (first) relatum than right ones. In Experiment 1c, we test the strength of this preference by manipulating the objects' visual salience.

### 2.3. Experiment 1c—perceptual salience: Flash

#### 2.3.1. Participants

Thirty nine native Dutch undergraduates from Tilburg University participated in this study for partial course credits. Data from 27 participants (18 women, mean age 20.3 years) were used, the rest being discarded on the basis of having noticed the cue (1 participant), task misunderstanding (2 participants) or not using a relatum at all as in *the ball in the center* (9 participants).

#### 2.3.2. Materials

Stimuli from Experiment 1b were used, slightly cropped so that the target object was placed exactly in the middle of the scene. The attention capture manipulation consisted of a black square, with an area of 0.5 × 0.5 degrees of visual angle, set against a white background (Gleitman et al., 2007).

#### 2.3.3. Procedure

The procedure was identical to the one presented in Experiment 1a. In addition, an implicit visual attention cue was added. Participants sat approximately 60 cm from the monitor, set to 1680 × 1050 pixels, 60 Hz refresh rate. Before each trial, participants were first presented with a fixation cross on a white background (500 ms). The fixation cross was followed by the attention capture manipulation, which was presented for 65 ms, followed immediately by a stimulus scene. The position on screen of the attention-capture cue varied (in half of the trials the cue was positioned left and in half right).

#### 2.3.4. Results and discussion

Participants used one of the two formulations (single-relatum *N* = 6, two-relata *N* = 21). Whether spatial position influenced reference production was tested by comparing the chance of mentioning the left entity as first relatum with random chance, using one–sample *t*-test. The chance of mentioning the left entity as first relatum was 67% [two-sided 95% CI [0.59, 0.75], *SD* = 0.19, *t*_(26)_ = 4.61, *p* < 0.001, *d* = 0.67].

Whether animacy or attention priming overruled the left bias was analyzed with an ANOVA test, having the Position of the Animate (2 levels: animate left, animate right) and the Position of the Flash (2 levels: flash left, flash right) as within subjects factors, and Participant Formulation Preference (2 levels: single-relatum, two-relata) as a between subjects factor. The ANOVA test revealed no statistically significant main effects of the Position of the Animate (*F* < 1) or of the Position of the Flash (*F* < 1).

There was a main effect of Participant Formulation Preference [*F*_(1, 25)_ = 6.66, *p* = 0.016, $\eta_{p}^{2}=0.21$]. In the two-relata formulation, participants mentioned more often the left entity as (first) relatum (*M* = 0.72), than in the single-relatum formulation (*M* = 0.51). There were no significant interactions between these factors (*F* < 1).

Experiment 1c confirmed the speaker's preference to mention left entities first. There were no effects of the Position of the Animate or of the Position of the Flash. In Experiment 1d, we continue testing the strength of the left bias by making one of the entities perceptually salient.

### 2.4. Experiment 1d—perceptual salience: Color

#### 2.4.1. Participants

Fifty five native Dutch undergraduates from Tilburg University participated in this study for partial course credits (32 women, mean age 22 years). One participant was discarded for never mentioning a relatum.

#### 2.4.2. Materials

**Figure 3:**
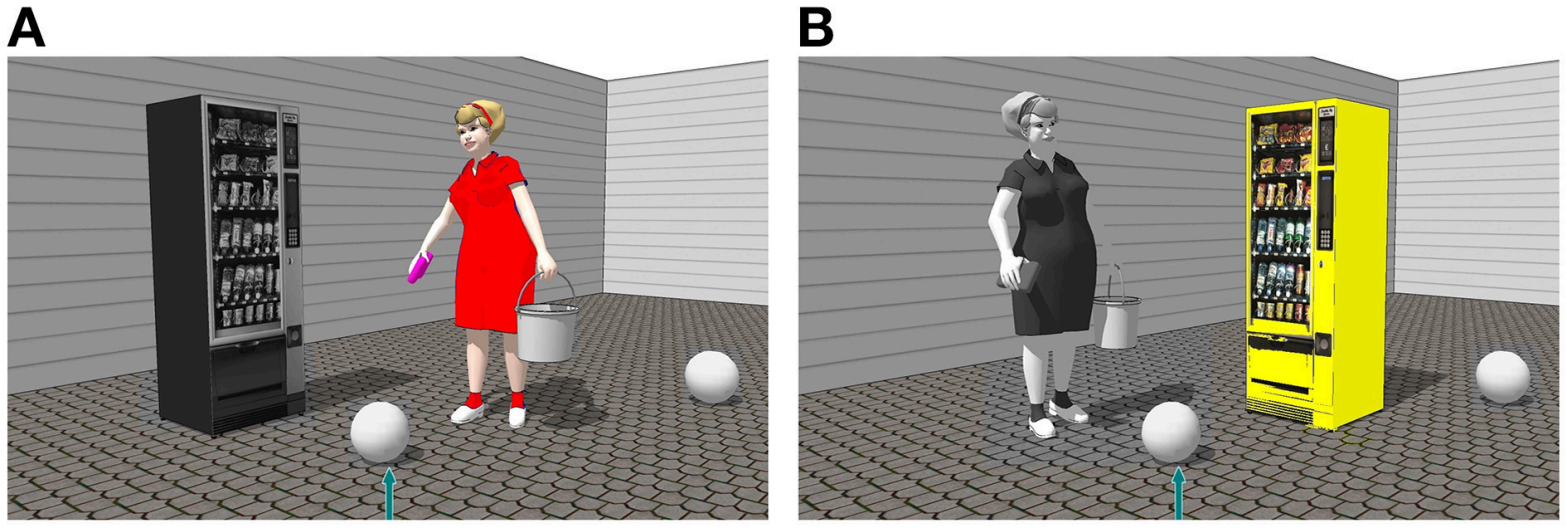
**Experimental stimulus with on the right of the target object in color (red) the animate object (A) and in color (yellow) inanimate object (B)**.

Stimuli from Experiment 1b were used. In addition, one relatum candidate in each picture had a unique color (red, blue, green or yellow), while all the other were grayscale (see Figure 3).

#### 2.4.3. Procedure

As in Experiment 1a. The position of the colored relatum candidate and the position of the relatum candidates was counterbalanced across presentation lists.

#### 2.4.4. Results and discussion

Participants used one of the two possible formulations (43 participants mentioned both relata, 4 participants mentioned a single relatum) or produced mixed descriptions across trials with both single-relatum and two-relata formulations (7 participants). Due to small sample sizes, participants that opted for a single-relatum were grouped with those who used a mixed formulation and analyzed as a mixed formulation group.

Whether spatial position influenced reference production was tested by comparing the chance of mentioning the left item as first relatum with random chance, using one–sample *t*-test. The chance of mentioning the left entity as first relatum was 61% [two-sided 95% CI [0.55, 0.66], *SD* = 0.20, *t*_(53)_ = 3.81, *p* < 0.001, *d* = 0.47].

Whether animacy or perceptual salience overruled the left bias was analyzed with an ANOVA test, having the Position of the Animate (2 levels: animate left, animate right) and the Position of the Colored entity (2 levels: colored left, colored right) as within subjects factors, and Participant Formulation Preference (2 levels: two-relata, mixed) as a between subjects factor.

There was no statistically significant effect of the Position of the Colored entity (*F* < 1).

There was a main effect of the Position of the Animate [*F*_(1, 52)_ = 18.645, *p* = 0.001, $\eta_{p}^{2}=0.264$]. Participants mentioned the left entity as relatum more often when the animate entity was placed on the right of the scene (*M* = 0.67) than when the animate was placed on the left (*M* = 0.43).

There was a main effect of Participant Formulation Preference [*F*_(1, 52)_ = 6.613, *p* = 0.01, $\eta_{p}^{2}=0.113$]. Participants mentioned the left entity as first relatum more often within a two-relata formulation (*M* = 0.63), than within a mixed one (*M* = 0.47).

There was an interaction between the Position of the Animate and Participant Formulation Preference [*F*_(1, 52)_ = 4.183, *p* < 0.05, $\eta_{p}^{2}=0.074$]. Speakers that used a two-relata formulation, mentioned the left entity as first relatum more often when the animate was on the right (*M* = 0.70) than on the left (*M* = 0.57). The same pattern of results was observed for speakers that used a mixed formulation (animate right *M* = 0.65, animate left *M* = 0.29). A split analysis showed that the general behavior of the two formulation groups is essentially the same, but the effect size is higher for the mixed formulation [*F*_(1, 10)_ = 7.101, *p* = 0.024, $\eta_{p}^{2}=0.415$], than for the two-relata one [*F*_(1, 42)_ = 7.809, *p* = 0.008, $\eta_{p}^{2}=0.157$].

Experiment 1d revealed that perceptual salience, namely entities with unique colors, did not influence reference production, while conceptual salience had a small influence.

Experiment 1 has examined the extent to which the production of spatial relational descriptions is influenced by spatial position and salience of potential relata. Our results showed that spatial position indeed influenced reference production: relatum objects positioned on the left in the scene were more likely to be mentioned as (first) relatum than those positioned on the right. However, participants did not systematically opt for the leftmost relatum object, suggesting that there might be other factors that could influence reference production as well. Therefore, in Experiments 1b–d, we manipulated the (conceptual and perceptual) salience of relatum objects, and these manipulations had no effect. In particular, we did not find that relatum objects that were salient, because of animacy, by priming visual attention or by using salient colors, were more likely to be used as (first) relatum. In Experiment 2, we assess if spatial position and salience affect listeners' evaluations of spatial descriptions.

## 3. Experiment 2—listener preferences

To further investigate the extent to which spatial position and salience might influence listeners' preferences for relata, in Experiment 2, participants were asked to rank relational descriptions. Given that many earlier studies have revealed strong effects of animacy, we expect descriptions that have an animate entity as (first) relatum to be ranked higher.

For pragmatic reasons, the language used in Experiment 2 was English. Earlier work on reference production (Theune et al., 2010; Koolen et al., 2012) suggested that English and Dutch are comparable in terms of the attributes used in descriptions.

### 3.1. Participants

Eighty-six English-speaking native participants from Australia, Canada and the UK were recruited via CrowdFlower, a crowdsourcing service similar to Amazon Mechanical Turk. The validity of this method for behavioral studies has been previously tested and studies assessing data quality have been positive about using crowdsourcing as an alternative to more traditional approaches of participant recruitment (e.g., Buhrmester et al., 2011; Crump et al., 2013). Ten participants' data were excluded for various reasons: because their ranking was identical (in more than 30% of the cases) to the order in which descriptions were presented (2 participants); because they declared being not native English speakers (5 participants); because did not finish the task (3 participants). The final sample included 66 participants (37 males, mean age 39.36 years, range 20–64 years).

### 3.2. Materials

The stimuli from Experiment 1b were used. The 32 experimental stimuli were divided across 6 randomized lists. The experiment consisted of 8 experimental stimuli (out of which 4 had an animate positioned left and 4 had an animate positioned right) and 8 filler stimuli. In addition, we used a set of four sentences representing the two participant formulation preferences using a single relatum and two relata. These sentences were translated from Dutch to English. The sentences were: *the ball in front of the ANIMATE* (e.g., the man); *the ball in front of the INANIMATE* (e.g., closet); *the ball between the ANIMATE and the INANIMATE*; *the ball between the INANIMATE and the ANIMATE*.

### 3.3. Procedure

First, participants were instructed to rank the four descriptions starting with the one they “liked best" given the visual scene. The descriptions were presented under each scene in random order. The participant could rank the descriptions by dragging them in an input field with four empty slots, where the slot no. 1 represented the description that participants liked most, while slot no. 4 was assigned for the description that they liked least. The picture remained on the screen until the participants had made their choice and pressed a button to continue. Each experimental trial was followed by one filler trial.

### 3.4. Results and discussion

For each trial, the order of the descriptions was ranked, starting from 1 (the best description) to 4 (the worst description).

Whether animacy influenced preferences was tested with a repeated measures ANOVA, having three within subjects factors: the Position of the Animate (2 levels: animate left, animate right), the Participant Formulation Preference (4 levels: in front of ANIMATE, in front of INANIMATE, between the ANIMATE and the INANIMATE, between the INANIMATE and the ANIMATE) and Scenes (4 levels)^2^.

**Figure 4:**
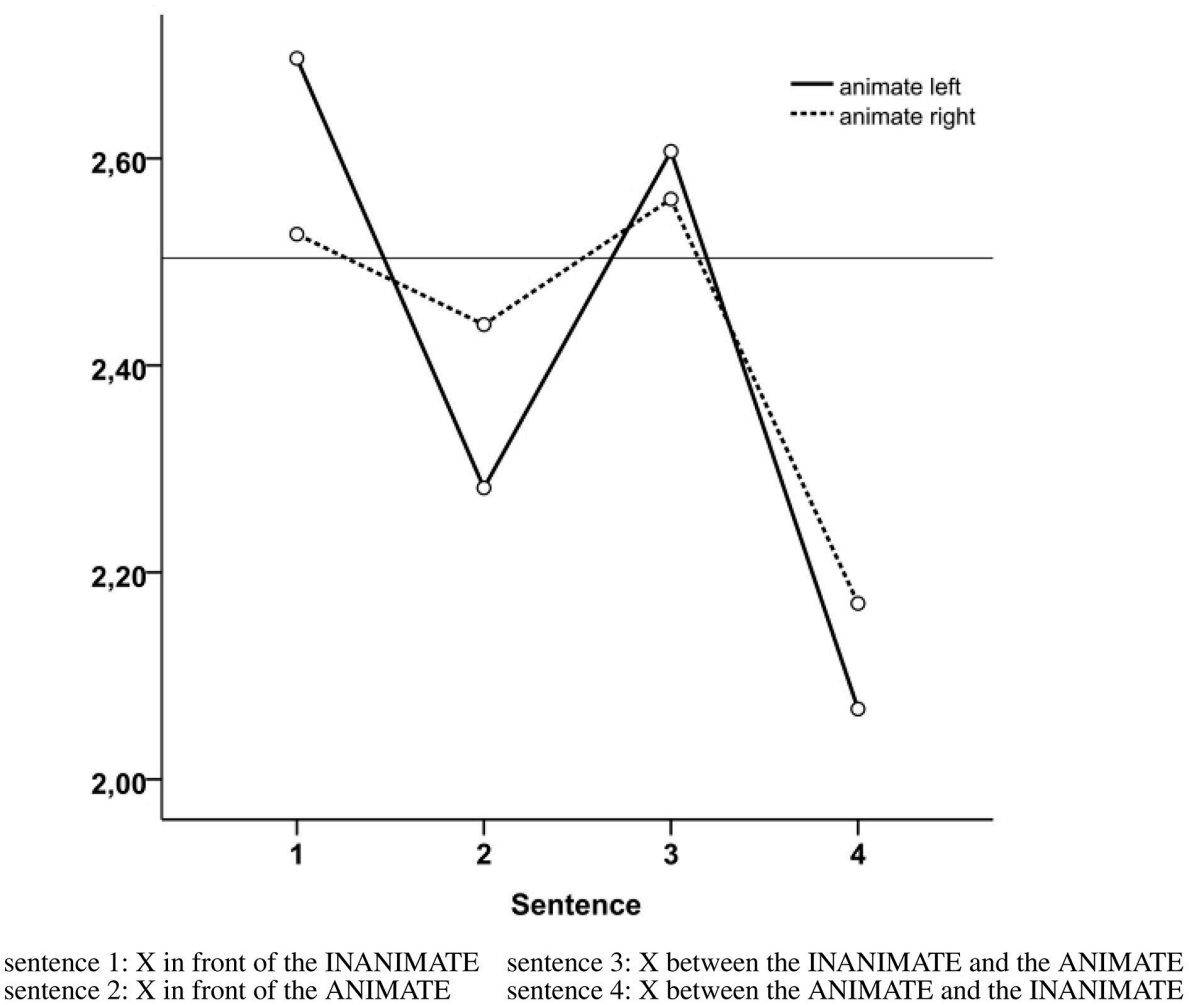
**Mean ranks across conditions (1 = highest preference, 4 = lowest preference), where 2.5 represents random chance**.

Results revealed a main effect of Participant Formulation Preference [*F*_(3, 306)_ = 5.186, *p* = 0.002, $\eta_{p}^{2}=0.048$] and a significant interaction between Animate Position and Participant Formulation Preference [*F*_(3, 306)_ = 4.412, *p* = 0.005, $\eta_{p}^{2}=0.041$]. Participants preferred the description that mentioned two relata and started with the animate irrespective of the visual scene (animate left *M* = 2.07, *SE* = 0.11; animate right *M* = 2.17 *SE* = 0.11) (see Figure 4). The second most preferred description was the one that mentioned a single relatum, namely the animate. This description was more preferred when the animate was positioned on the left of the scene (*M* = 2.28, *SE* = 0.08) than on the right of the scene [*M* = 2.44, *SE* = 0.09; *F*_(1, 102)_ = 6.58, *p* = 0.003, $\eta_{p}^{2}=0.082$]. The least preferred description was the one mentioning a single inanimate relatum, especially when the animate was placed on the left [*M* = 2.70, *SE* = 0.09; animate placed right *M* = 2.53, *SE* = 0.09; *F*_(1, 102)_ = 9.08, *p* = 0.012, $\eta_{p}^{2}=0.061$].

## 4. Conclusions and discussion

The main aim of this study was to examine the extent to which production of spatial relational descriptions is influenced by spatial position and salience. Our results show that spatial position systematically influenced reference production. A basic language elicitation task determined that speakers often mentioned the entity positioned leftmost in the scene as (first) relatum. This was consistent across four production experiments (highest mean 67%, $\eta_{p}^{2}$ range 0.47–0.75). Based on these observations, we considered that other factors might influence reference production. Thus, we investigated possible effects of the objects' (conceptual and perceptual) salience. In Experiment 1b, conceptual salience was manipulated visually, by having an animate and an inanimate relatum candidate. Despite the strong body of research arguing for effects of animacy in reference production, animacy was found to have a significant effect in only one out of three production studies (Experiment 1d). Visual salience was manipulated using two different methods. In Experiment 1c, attention was primed using a flash and in Experiment 1d, the objects were made perceptually salient by having a distinctive color. These manipulations yielded no effects. From a listener's perspective, the formulation of the description and the position of the animate entity in the scene influenced to some extent the acceptability rating (Experiment 2). These results are further discussed in relation to broader aspects of reference production.

### 4.1. Relevance for reference production

The studies reported bring evidence for relatum reference being influenced by the inherent spatial structure of the scene, a factor largely unexplored in studies of (computational) reference production. Across different circumstances, there was a systematic preference for mentioning left entities as (first) relatum in relational descriptions such as *in front of X; in between X and Y*. This preference could have been caused either by cultural differences or spatial asymmetries in scene scanning. It is worth replicating Experiment 1 with speakers of a language with a right-to-left system.

The position of the object seems to be a constant factor influencing reference production. Our results are consistent with Miller et al. (2011), who stress that the spatial relation between the target and the relatum candidates is an important predictor in relatum selection. Congruent evidence comes from Clarke et al. (2013b), who report that position (measured in relation to the center of the screen) contributes to perceptual salience of the object and affects the likelihood with which objects are mentioned. When objects are symmetrically arranged, not only spatial position, but also salience influence (to some extent) referential choices.

Previous research has granted an important role to salience in reference production. Visually salient and linguistically important (e.g., animate) objects are more likely to be mentioned, as well as objects spatially placed in a prominent position Clarke et al. (2013b). In these studies, we have manipulated salience on conceptual and visual levels. We expected salient entities to influence the ordering of linguistic elements in the spatial relation and be mentioned (first) more often than the other candidates. Surprisingly, there were poor effects of animacy, no effects of the visual salience manipulation. Below we address a few questions related to these results.

First, why did animacy have a limited influence on reference? The impact of animacy on word order, and more precisely on conjunctive phrases is debatable (see Branigan et al., 2008). For example, when the conjoined NPs are presented embedded in a sentence such as *the dog and the telephone were making noise* or *the surgeon yelled for a nurse and a needle* (experiments 1 and 2 in McDonald et al., 1993), animacy had no reliable effect on conjunct order. However, when removed from sentences and produced in isolated phrases (experiments 3, 4, and 5 in McDonald et al., 1993), animate nouns regularly occupied a leading position. It is conceivable that the effect of animacy in the current studies might have been dampened by sentence context, in line with the findings of McDonald et al. (1993). Compared to other experiments that found a strong effect of animacy on reference production in visual domains (e.g., Coco and Keller, 2009), in our studies animacy was manipulated visually, without priming participants with animacy in a lexical format. “Visual animacy” was suggested to be a less important factor in attention guiding (Wolfe and Horowitz, 2004). Interestingly, the results of the acceptability rating task (Experiment 2) present a different picture, which is more in line with previous studies suggesting strong effects of animacy and is in apparent contrast with the production data from Experiment 1. Descriptions which included an animate entity as the first (or the only) relatum were rated higher than those having an inanimate as first or single relatum. In fact, the descriptions which had animate as first relatum were rated as the most acceptable, irrespective of the spatial placement of the objects in the scene. Not only animacy, but also the left bias seemed to have influenced the acceptability ratings, as descriptions containing a single animate relatum, were rated higher when the animate entity was placed on the left, rather than on the right side of the visual scene and the same pattern was observed for descriptions that included a single inanimate relatum. This slight discrepancy between the results of Experiments 1, 2 highlights an observation that has been made before in the context of REG evaluation: what speakers do is not necessarily what is appreciated most by addressees (for a review, see Gatt and Belz, 2010; Krahmer and Van Deemter, 2012).

Second, why did priming attention have no effect? Directing speakers' attention to a specific region of the scene predicts which entity would be mentioned first, both in sentences and in conjoined NP descriptions (Gleitman et al., 2007). Yet, in our study, the attention capture cue did not influence utterances. Preference for left entities was stable, even when visual attention was directed to a different relatum candidate. It might be the case that the effect of the cue fades during production (the first-mentioned entity in our scenario was always the target ball). Other studies also report no effect of this attention priming manipulation (Nappa and Arnold, 2014; Arnold and Lao, 2015). In addition, when salience was explicitly manipulated by making an object perceptually salient, it did not yield a significant effect. This might be caused by the visual simplicity of the stimuli.

The extent to which our results can be observed using complex visual scenes also warrants further study. For example, Viethen and Dale (2008) reported (limited) effects of relatum salience in scenes consisting of three objects with simple spatial arrangements, but in a more complex study, salient large relata did not systematically influence whether the object was mentioned or not (Viethen et al., 2011). Similarly, participants describing routes through groups of colored objects in a MapTask (Louwerse et al., 2007) seem to have disregarded potential visual distractors (Viethen and Dale, 2011). The results of Elsner et al. (2014); Clarke et al. (2013a) reveal a different picture: in very cluttered and complex scenes, like the Where's Wally pictures, speakers were sensitive to perceptual salience, not only when choosing the objects to mention, but also when producing a description. The relational descriptions started more often with the salient object. Nonetheless, our studies are complementary, showing (though to a smaller extent) effects of the position an object occupies in the scene and salience.

Our experiments have a number of limitations. As mentioned above the scenes used as stimuli were simple and consisted of a small number of objects. Ideally, future research should take into account scenes of a higher visual complexity, use a different spatial arrangement of the objects and manipulate other perceptual features (such as size) as well. For a systematic analysis other tasks should be considered as well (e.g., testing listeners' comprehension in a reaction time study).

In the production experiment, we also discouraged participants from saying “the ball on the left.” While objects in visual environments can be referred to with a wide variety of forms of spatial language, we wanted to focus on referential choices when describing objects in relations. However, we also acknowledge that identifying a target by mentioning its location (and thus, maybe contrasting the target with a potential distractor, see Tenbrink, 2005) is a widespread strategy. Crucially, more research is needed to find out when people need or prefer relational descriptions containing explicit relata.

### 4.2. Formulation preferences

As for the formulations used, across studies, a small sample of participants chose a single relatum, thus producing a *X in front of Y* description. The chance of choosing one of the entities was not influenced by the distance between the relatum and the position of the distractor (the further away the relatum object was from the distractor ball, the less ambiguous).

Most of the participants referred to the target using the preposition *tussen* (in between), which describes the location of the target in relation to both relata. Compared with other locative prepositions, *in between* is a syntactically complex and cognitively more expensive one (because it contains more words and involves more relata), but it also provides a more accurate description. This preposition might be preferred due to the view point from which the speaker looks at the scene (Kelleher et al., 2010), from which the relatum candidates and the target seem arranged in an almost linear fashion. In fact, when the target object is situated between two other elements and the in between relation is available for reference, speakers will often use this option (Tenbrink, 2007, p.261).

### 4.3. Recommendations for referring expressions algorithms

Understanding the criteria on which humans base their referential choices offers insights for the development of referring expressions generation algorithms. There are only few algorithms that make use of extrinsic attributes as a last resort (e.g., Dale and Haddock, 1991; Gardent, 2002; Krahmer and Theune, 2002; Krahmer et al., 2003; Varges, 2005). Crucially, more research is needed to find out when people need or prefer relational descriptions containing explicit relata. Nevertheless, these systems have little to say about relatum reference as they assume access to a predefined scene model, where the relata has been selected and treat spatial reference as the last means for generating a unique description. Though there are some assumptions regarding the factors that drive choices regarding relatum reference, there is no systematic research on this issue. For example, Krahmer and Theune (2002) note that human speakers and hearers might have a preference for relata which are close to the target. Kelleher et al. (2005) implement a measure for proximity and bring into discussion visual and discourse salience. Dos Santos Silva and Paraboni (2015) consider distance as the main factor, followed by the unique spatial relations between objects. Apart from distance, various other factors may influence relatum reference. For example, Elsner et al. (2014) highlight that visual features that contribute to the object's perceptual salience should be taken into account in order to generate more human-like reference in visual domains. Specifically, perceptual salience (spatial and visual information) influences the order in which relata are mentioned in relational descriptions.

Our results suggest that algorithms should take into account the spatial position and the object's salience. When the distance between target and the relatum candidates is similar, the spatial structure of the scene should be the first feature to be examined. In circumstances in which there are several relatum candidates similarly aligned, we suggest that entities placed on the left of the target to be favored. Perceptual and conceptual salience might also be taken into account. Given the practical nature of REG, the human-likeness aspect should be balanced with a comprehension-oriented perspective (e.g., Paraboni et al., 2007; Garoufi, 2013; Mast et al., 2014). Our results suggest that if the goal of the system is different from just producing a human-like expression, other factors might play a role (see also Krahmer and Van Deemter, 2012). More addressee oriented (and maybe more efficient) descriptions might be produced when including an animate as first relatum. Our results suggest that when the target object is situated between two other objects and the *in between* relation is available for reference, the system should refer to both objects and start with the animate irrespective of the position of the objects in the scene. However, if the system generates a description with a single relatum, this relatum preferably should be the object located on the left of the target.

Finally, speakers have to make several referential choices when uttering spatial descriptions and different factors can influence this process. The results of this study suggest that reference production was affected by the spatial position of a relatum candidate and less so by (conceptual and perceptual) salience.

## Author contributions

All authors listed, have made substantial, direct and intellectual contribution to the work, and approved it for publication.

## Funding

The first author received financial support from the Netherlands Organization for Scientific Research (NWO), Promoties in Geesteswetenschappen (322-89-008), which is greatly acknowledged.

### Conflict of interest statement

The authors declare that the research was conducted in the absence of any commercial or financial relationships that could be construed as a potential conflict of interest.

## Footnotes

^1^The Huynh-Feldt epsilon value was pretty close to 1 in all the analyses, indicating that there was no need for adjustments of the degrees of freedom.

^2^The analyses were also done using non-parametric Friedman's signed rank tests which yielded similar results.
